# Why vary what’s working? Phase variation and biofilm formation in *Francisella tularensis*

**DOI:** 10.3389/fmicb.2022.1076694

**Published:** 2022-12-06

**Authors:** Kevin D. Mlynek, Joel A. Bozue

**Affiliations:** Bacteriology Division, U.S. Army Medical Research Institute of Infectious Diseases (USAMRIID), Frederick, MD, United States

**Keywords:** *Francisella tularensis*, phase variation, biofilm, O-antigen, host adaptation, environmental survival, vectors and bacteria, VBNC (viable, but non-culturable)

## Abstract

The notoriety of high-consequence human pathogens has increased in recent years and, rightfully, research efforts have focused on understanding host-pathogen interactions. *Francisella tularensis* has been detected in an impressively broad range of vertebrate hosts as well as numerous arthropod vectors and single-celled organisms. Two clinically important subspecies, *F. tularensis* subsp. *tularensis* (Type A) and *F. tularensis* subsp. *holarctica* (Type B), are responsible for the majority of tularemia cases in humans. The success of this bacterium in mammalian hosts can be at least partly attributed to a unique LPS molecule that allows the bacterium to avoid detection by the host immune system. Curiously, phase variation of the O-antigen incorporated into LPS has been documented in these subspecies of *F. tularensis,* and these variants often display some level of attenuation in infection models. While the role of phase variation in *F. tularensis* biology is unclear, it has been suggested that this phenomenon can aid in environmental survival and persistence. Biofilms have been established as the predominant lifestyle of many bacteria in the environment, though, it was previously thought that Type A and B isolates of *F. tularensis* typically form poor biofilms. Recent studies question this ideology as it was shown that alteration of the O-antigen allows robust biofilm formation in both Type A and B isolates. This review aims to explore the link between phase variation of the O-antigen, biofilm formation, and environmental persistence with an emphasis on clinically relevant subspecies and how understanding these poorly studied mechanisms could lead to new medical countermeasures to combat tularemia.

## Introduction

Found ubiquitously across the Northern Hemisphere, *Francisella tularensis* is the etiological agent of tularemia, or more casually, “rabbit fever”(Ellis et al., 2002). In humans, illness presents in several forms with the most common being ulceroglandular, often from the bite of an insect carrying *F. tularensis* or contact with an infected rabbit. From a biodefense perspective, tularemia could also manifest as the more severe form of the disease-causing pneumonic or typhoidal tularemia (Ellis et al., 2002). *F. tularensis* is comprised of two subspecies that cause human disease: *F. tularensis* subsp. *tularensis* (Type A) and *F. tularensis* subsp. *holarctica* (Type B). A third closely related subspecies, *F. tularensis* subsp. *novicida*, is only associated with brackish water or soil and rarely causes disease in humans. *F. novicida* is commonly used as a laboratory surrogate since it has a high degree of genetic similarity to *F. tularensis*, is able to infect macrophages *in vitro* and cause disease in mice, and can be handled under BSL-2 conditions (Kingry and Petersen, 2014). Throughout this manuscript, *F. novicida* will be used to note this subspecies exclusively while *F. tularensis* will be used to describe Type A and B isolates specifically.

*Francisella tularensis* is classified as a Tier 1 Select Agent in the United States (Dennis et al., 2001) and currently no approved Food and Drug Administration (FDA) vaccine is available. While a Live Vaccine Strain (LVS) exists and has been used for laboratory workers at risk in the past under Investigational New Drug (IND) status, the basis of attenuation is poorly characterized and the ancestral strain is unknown(Eigelsbach and Downs, 1961; Saslaw et al., 1961; Hornick and Eigelsbach, 1966). Complicating the approval of LVS is spontaneous variation of colony phenotypes, which is known to adversely affect immunization (Eigelsbach et al., 1951; Eigelsbach and Downs, 1961). Early studies determined that the frequency of colony variation, referred to as phase or blue/gray variation, could be as high as 10^−3^ to 10^−4^ depending on culture conditions and, paradoxically, variant colony phenotypes dramatically differed in terms of virulence when tested in a murine model (Eigelsbach et al., 1951). With this in mind, it is curious that the mechanisms responsible for this variation were not naturally selected against given the consequence for attenuation of an intracellular pathogen. Furthermore, understanding how this variation occurs in *F. tularensis* could lead to ways to prevent phase variation from occurring and allow for a more stable and efficacious live vaccine.

Biofilm refers to an adhered community of cells encased by an extracellular matrix (ECM) and typically involves distinct changes in bacterial behavior, gene expression, and metabolism that are not observed in the planktonic state (Hall-Stoodley et al., 2004). In pathogens, biofilm is often regarded as a virulence determinant as it enables bacteria to cope with the host environment by thwarting innate immunity, phagocytosis, and antibiotic treatment (Hall-Stoodley and Stoodley, 2009). Francisella species have been shown to form biofilm [reviewed by van Hoek (2013)], though clinically important subspecies of *F. tularensis* tend to form a less defined biofilm with sparse cell density than *F. novicida* or other Francisella species(Margolis et al., 2010; Mahajan et al., 2011). Recent studies demonstrate that O-antigen (O-Ag) of the LPS can influence the biofilm-forming capacity of Type A and B isolates, changing this perception of biofilm formation in *F. tularensis* (Champion et al., 2019; Mlynek et al., 2021). This review aims to explore where phase variation and biofilm may play a role in the survival and pathogenesis of *F. tularensis* which could lead to new therapies to prevent disease.

### Comparing apples to oranges: Distinct genetic differences between *Francisella novicida* and *Francisella tularensis* complicate understanding biofilm formation in clinically relevant isolates

While *F. novicida* shares a high degree of genetic similarity with *F. tularensis* for which it has been utilized as a surrogate strain, important genetic differences do exist. First, *F. tularensis* lacks a functional cyclic-di-GMP system (cd-GMP) which is present in *F. novicida*. Secondly, the *wbt* locus of *F. tularensis* (involved in O-Ag synthesis) contains additional genes that are not present in *F. novicida* (McLendon et al., 2006; Sjodin et al., 2012; Kingry and Petersen, 2014). These differences between *F. novicida* and the virulent *F. tularensis* strains highlight the need to focus on the latter pathogenic strains to gain a true understanding of biofilm and variation.

cd-GMP has been shown to be an important second messenger that impacts multiple aspects of bacterial behavior, often influencing genes responsible for the transition between the environment and hosts in pathogenic species (Tamayo et al., 2007; Jenal et al., 2017). The model that has emerged is that elevated levels of cd-GMP inhibit motility and stimulate biofilm formation by downregulation of the genes encoding proteins required for flagella and/or pili and upregulation of genes encoding extracellular polysaccharide (Simm et al., 2004; Hickman et al., 2005). The intracellular concentrations of cd-GMP are tightly controlled by diguanylate cyclase (DGC; for synthesis) and phosphodiesterase enzymes (PDE; for degradation). The activity of these enzymes depends on a myriad of input signals, often through the interaction with other proteins, such as two-component systems (Galperin et al., 2001). The gene cluster responsible for modulating the levels of cd-GMP in *F. novicida* (FTN_ 0451 to FTN_0456) was noted to be absent in both LVS and Schu S4 (Rohmer et al., 2007) and appears to be absent or incomplete in all *F. tularensis* genomes published to date. While it is unclear if other genes encode DGC/PDE enzymes capable of modulating cd-GMP levels in *F. tularensis*, cd-GMP appears to control virulence and biofilm in *F. novicida* in a similar manner to what has been reported in other bacterial pathogens. A study by Zogaj et al., demonstrated that high cd-GMP levels tempered virulence of *F. novicida* by inhibiting intracellular replication and promoted biofilm formation (Zogaj et al., 2012). While Francisella genomes have a limited repertoire of *bona fide* two-component systems, it was also found that the orphaned response regulator QseB influenced DGC activity, which is consistent with other reports suggesting a role for QseB in *F. novicida* biofilm formation (Durham-Colleran et al., 2010; Zogaj et al., 2012). However, mechanisms responsible for the regulation of biofilm formation in Type A and B isolates are largely unknown.

LPS is considered a major virulence factor in *F. tularensis* as it allows the bacterial cell to evade the host immune response (Sandstrom et al., 1992; Gunn and Ernst, 2007; Wang et al., 2007; Weiss et al., 2007) and deleterious mutations within genes involved in LPS or O-Ag synthesis often lead to attenuation (Raynaud et al., 2007; Apicella et al., 2010; Kim et al., 2012; Jones et al., 2014; Rasmussen et al., 2014, 2015; Chance et al., 2017). While the structure of *F. tularensis* LPS contains notable and unique features for lipid A, core, and O-Ag [reviewed by Gunn and Ernst, 2007], recent studies suggest the O-Ag is a driving determinant of biofilm-forming capacity (Champion et al., 2019; Mlynek et al., 2021). As the case with a wide range of other Gram-negative bacteria, O-Ag has been demonstrated to play a role in biofilm formation (Nakao et al., 2006, 2012; Murphy et al., 2014; Hathroubi et al., 2016).

*Francisella tularensis* isolates share an O-Ag repeat of Qui4NFm-GalNAcAN-GalNAcAN-GuiNac (Vinogradov et al., 1991, 2002; Prior et al., 2003). In contrast, the terminal saccharides differ in *F. novicida* with the QuiNAc4NAc at the reducing residue and a third GalNAcAN at the non-reducing residue (Vinogradov et al., 2004; Thomas et al., 2007). The most likely cause for this difference is that *F. tularensis* contains five additional genes (*wbtI, wbtJ, wbtK, wbtL,* and *wbtM*) within the *wbt* locus (McLendon et al., 2006; Rohmer et al., 2007; Sjodin et al., 2012). Each additional gene is thought to encode for an enzyme involved in the biosynthesis of Qui4NFm (Prior et al., 2003; Li et al., 2007; Twine et al., 2012; Zimmer et al., 2014). Furthermore, mutations made to genes within the *wbt* operon can result in an altered O-Ag phenotype consistent with “phase” or “gray” variants (Bandara et al., 2011). Interestingly, growth environment can influence the relative amount of O-Ag displayed on the cell surface, either by LPS chain length or incorporation into a capsule, suggesting that this feature may be important in host or environmental adaptation (Zarrella et al., 2011; Holland et al., 2017). However, transcriptional regulation of the O-Ag has not been associated with phase variation as this phenomenon is attributed to physical alterations of this molecule (Cowley et al., 1996; Hartley et al., 2006; Soni et al., 2010).

### O-antigen influences the biofilm-forming capacity of *Francisella tularensis* isolates

Early researchers noted a distinct reduction in virulence when *F. tularensis* was cultured on artificial medium (Foshay, 1932; Ransmeier, 1943; Eigelsbach et al., 1951). A study by Eigelsbach in 1951 aimed to systematically correlate colony morphology to virulence by identifying smooth and non-smooth colonies and then further classifying these colonies using oblique lighting. It was found that medium pH, inoculum size, and culture duration influenced the rate at which spontaneous changes in colony morphology occurred. Most importantly, this study determined that colonies which appeared gray under oblique lighting were highly attenuated compared to those that appeared blue. Eigelsbach also noted that some of these morphologies were unstable, and reversion between blue and gray variant forms was possible. It would be 45 years before these findings were linked to an antigenic shift of the LPS O-Ag displayed on the cell surface (Cowley et al., 1996).

A study by Champion et al. identified O-Ag and glycosylation of the capsule-like complex (CLC) as factors that can influence biofilm formation in *F. tularensis* isolates (Champion et al., 2019). While *F. novicida* can form a robust biofilm in 2–3 days, *F. tularensis* isolates typically required 10 days to form a comparable biofilm. O-Ag deficient mutants of LVS and TI0902 (virulent Type A) developed biofilms within 5 days that were 2-5x more robust than parental strains (Champion et al., 2019). Further, surface attachment and biofilm formation were enhanced in a double mutant deficient in O-Ag and CLC glycosylation, suggesting that there is an inverse relationship between cell surface carbohydrates and biofilm formation. It is notable that *F. tularensis* can produce an electron transparent CLC as well as an electron-dense capsule, with the latter being chiefly composed of O-Ag [Hood, 1977; Cherwonogrodzky et al., 1994; Apicella et al., 2010; reviewed by Freudenberger Catanzaro and Inzana, 2020]. Highlighting the differences in biofilm formation among *F. tularensis* subspecies, O-Ag mutants in *F. novicida* form biofilms that are equal or less than the parental wild-type (Champion et al., 2019).

Mlynek et al. identified a link between variation of the O-Ag and biofilm formation in *F. tularensis* (Mlynek et al., 2021). In agreement with the Champion findings, biofilm formation typically required at least 7 days to occur. However, it was noted that robust biofilm formation occurred stochastically at earlier time points and increased in frequency as culture duration increased. It was subsequently determined that biofilm formation was associated with a distinct population of gray variants that emerged within the culture. While the exact alteration of the LPS in these variants was unknown, western blotting with α-LPS or α-capsule mAbs against O-Ag yielded no reactivity (Mlynek et al., 2021). The studies Champion et al. and Mlynek et al. found that external conditions, such as culture medium and pH, impacted biofilm development, which suggests additional gene regulation and/or environmental checkpoints beyond antigenic variation of O-Ag may factor into *F. tularensis* biofilm formation.

### Is variety the spice of life for *Francisella tularensis*?

**Figure 1:**
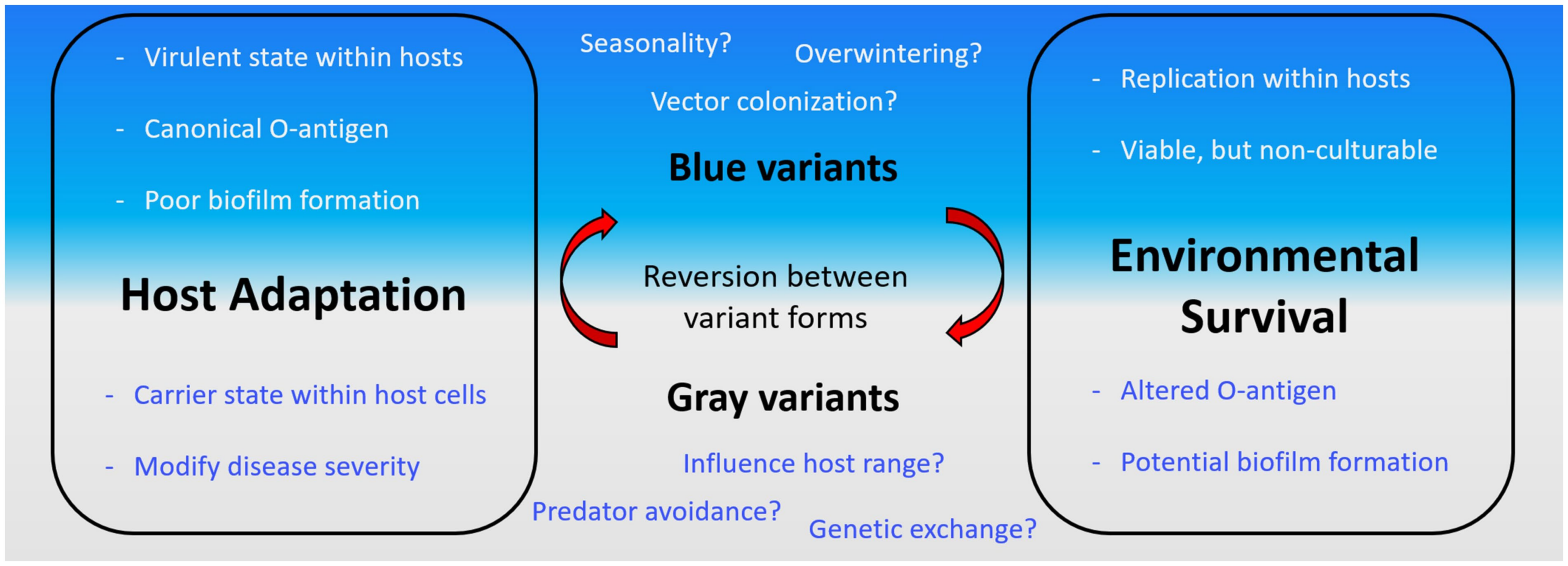
Traits of *Francisella tularensis* antigenic variation and outstanding questions. *F. tularensis* is known to transition between a “blue” or “gray” state by altering the O-antigen. The properties of these states are outlined in each box (Host Adaptation, left; Environmental Survival, right) grouped by where trait likely aids the bacterial cell. The traits are further separated within these boxes based on the associated state of the cell (blue, top; gray, bottom). Outstanding aspects of *F. tularensis* biology where phase variation is potentially important are displayed in blue or gray text.

*F. tularensis* has been identified in a diverse collection of hosts ranging from amoeba to humans. Further, it has been well established that *F. tularensis* can be harbored within arthropod vectors as well as detected in water sources. While biofilm is typically thought to be the predominant lifestyle of bacteria in the environment, it is unclear what role, if any, biofilm plays in these diverse environments and how phase variation, or more properly, antigenic variation factors into biofilm formation by *F. tularensis* in this context (Figure 1).

#### Vertebrates and hosts cells

Given the pathogenicity of this bacterium for humans, research efforts have focused on the mammalian host cell response and the role of LPS in avoiding immune detection. While structural modifications to lipid A and core of the LPS have been attributed to phase variation (Soni et al., 2010), the most common cause of phase variation is arguably alterations to the O-Ag (Cowley et al., 1996; Hartley et al., 2006). In Francisella, it has been well established that O-Ag is important for the subversion of mammalian innate immune defenses, namely complement (Sandstrom et al., 1988; Sorokin et al., 1996; Ben Nasr and Klimpel, 2008; Clay et al., 2008) and, along these lines, serum increases the uptake of gray variants by macrophages (Hartley et al., 2006). Additionally, poor intracellular replication of gray variants within macrophages has been reported (Thomas et al., 2007; Lindemann et al., 2011). However, this begs the question why is phase variation frequently observed in *F. tularensis* if immune evasion and intracellular replication are decreased in variants?

One hypothesis is that *F. tularensis* gray variants (GVs) enable persistence inside host macrophages by stimulating bacteriostatic levels of the nitric oxide response to set up a carrier state (Cowley et al., 1996). Recently, biofilm-like structures, termed intracellular bacterial communities (IBC), have been identified for many bacteria persisting intracellularly within host cells [reviewed by Mirzaei et al., 2020]. IBCs consist of small aggregates of cells encased within a matrix, often formed from extracellular polysaccharides and/or pili. It is currently unclear if GVs produce biofilm when grown intracellularly, though conceivably, IBCs would contribute to persistence within the host. While *F. tularensis* is typically studied as an intracellular pathogen, bacteria can be detected extracellularly in host blood (Forestal et al., 2007; Yu et al., 2008). In this environment, biofilm could play a more traditional role in pathogenesis and armor variants during the extracellular phase of the infection. Outer membrane vesicles (OMV) have been shown to stimulate biofilm in *F. tularensis* (Siebert et al., 2019) and facilitate early interactions with macrophages (McCaig et al., 2013; Pavkova et al., 2021); however, OMVs were not detected when the bacterium is intracellular (Pavkova et al., 2021).

Alternatively, if GVs and by extension biofilm formation are important for *F. tularensis* pathogenesis, phase variation may impede the ability to survive within certain hosts which has implications for host range. Supporting this hypothesis, many mammalian animal models are highly susceptible to *F. tularensis*, especially at low doses (<10 CFU), while only mice typically succumb to *F. novicida* at this dose [reviewed in Kingry and Petersen, 2014]. The LPS structure is likely a contributing factor to differences in host susceptibility as *F. tularensis* growth was suppressed in rat macrophages by the nitric oxide response when co-infected with *F. novicida* (Cowley et al., 1997). While *F. tularensis* infects a plethora of vertebrate hosts (including, amphibian, fish, birds, and mammals; Burroughs et al., 1945; Hopla, 1974), it is largely unknown how the immune response of individual species reacts to *F. tularensis* LPS and phase variant O-Ag. It is possible that the LPS presented by GVs favors the survival of *F. tularensis* in vertebrate host other than routinely studied laboratory models. Further studies are needed to address the *in vivo* role of biofilm formation during vertebrate infections in *F. tularensis*.

#### Arthropod vectors

Arthropod transmission of *F. tularensis* to humans has been noted nearly since the first reports of tularemia in the early 1900s (Francis, 1919; Parker et al., 1924), and still today, the glandular and ulceroglandular forms are by far the most common manifestation of tularemia in humans (Rosenberg et al., 2018). Association with a vector can increase survivorship and biological transmission of a bacterial population while decreasing the risk of “dead-end” infections in pathogens associated with a high mortality. Biofilm has been found to aid in the transmission of bacterial pathogens closely associated with arthropod vectors to mammalian hosts with the most notable example being *Yersinia pestis* in fleas (Jarrett et al., 2004; Hinnebusch and Erickson, 2008). While *F. tularensis* is transmissible through multiple vectors, tularemia is mostly associated with bites from deer flies, mosquitoes, and hard ticks [reviewed in Telford and Goethert (2020)].

Type A and B isolates tend to have different vector ecology. Classically, Type A isolates have been associated with ticks in arid environments, while Type B isolates have more often been associated with mosquitos near aquatic systems (Parker et al., 1951; Jellison, 1974; Eliasson et al., 2006; Tully and Huntley, 2020). Transstadial transmission has been reported for both vectors (Lundstrom et al., 2011; Backman et al., 2015; Coburn et al., 2015; Mani et al., 2015), with ticks maintaining bacteria capable of infection at each stage (Coburn et al., 2015; Mani et al., 2015) and the American dog tick (*Dermacentor variabilis*) was identified as a major factor for perpetuation in an environmental setting (Goethert et al., 2004; Goethert and Telford, 2009). While a fair amount of literature has been published on the ticks and tularemia (thorough reviews by the Huntley group; Zellner and Huntley, 2019; Tully and Huntley, 2020), little is known about the role, if any, of *F. tularensis* biofilm formation in tick vectors.

Mosquito larvae have been shown to feed on both planktonic and biofilm cultures of *F. tularensis* in an aquatic laboratory setting (Mahajan et al., 2011; Backman et al., 2015). In these models, *F. tularensis* localized intracellularly within the mosquito, suggesting that bacterial persistence in this vector is not solely due to ingestion. In this context, the seasonality of tularemia in mammals could be indicative of an overwintering state within arthropod vectors; however, long-term exposure to *F. tularensis* biofilm lowered the overall fitness of mosquito larvae and fecundity of adults (Mahajan et al., 2011).

An important aspect to note is that invertebrate immunology remains largely unknown in these vectors, making it difficult to interpret if phase variation or biofilm is advantageous for survival within arthropods. Drosophila have been shown to detect LPS through C-type lectins and β-glucan recognition proteins (βGRPs; Kim et al., 2000; Xia et al., 2018). A careful examination of the role biofilm formation plays in arthropod vectors, if any, would greatly advance the understanding of both biofilm and LPS variation.

### Protozoan hosts and aquatic survival

The presence of *F. tularensis* in aquatic systems places the bacterium in proximity of single-celled organisms, such as amoeba (Hopla, 1974; Abd et al., 2003), as well as an environment that provides the potential for multi-species biofilms. It is possible that both phase variation and biofilm could offer a competitive advantage. For instance, it has been shown that protozoan preying upon *Salmonella* display feeding preference when presented multiple serovars and can distinguish prey based solely upon the O-Ag (Wildschutte et al., 2004). Along these lines, phase variation may enable predator avoidance as the edibility of *F. tularensis* has previously been shown to differ depending on subspecies, even isolate (Thelaus et al., 2009). However, Abd et al. demonstrated that *F. tularensis* can utilize *Acanthamoeba castellanii* as a host, following an infection cycle that is similar to murine macrophages once bacteria were engulfed (Anthony et al., 1991; Abd et al., 2003; El-Etr et al., 2009). Further work is needed to determine if protozoans display preference for a particular *F. tularensis* O-Ag variant as well as the mechanisms enabling invasion.

*Francisella tularensis* has been found to remain viable as determined by CFU counts in conditions mimicking natural water for at least 3 weeks (Golovliov et al., 2021). Under these conditions, wild-type *F. tularensis* strains did not form biofilms; however, a *wbtI* mutant (O-Ag deficient) formed biofilms that were maintained throughout the study at both 20°C and 4°C (Golovliov et al., 2021). Differences in virulence were observed after 24 weeks at 4°C, as mice infected with Schu S4 (Type A) displayed no symptoms of disease, while FSC200 (Type B) remained virulent (Golovliov et al., 2021). Along these lines, LVS was found to persist in a viable, but non-culturable state (VBNC) for at least 140 days at 8°C in tap water, but at the cost of virulence as mice were symptomless when challenged with VBNC cells (Forsman et al., 2000). A connection between biofilm-forming variants and a VBNC state was found by Mlynek et al. (2021), but it is unclear how *F. tularensis* prioritizes the convergence of mechanisms that could impact environmental survival. External signals such as nutrient availability or pH have been found to factor into both phase variation and biofilm formation (Eigelsbach et al., 1951; Champion et al., 2019; Mlynek et al., 2021).

### Additional considerations

Biofilm formation is considered a virulence determinant in many bacteria as it facilitates the establishment of chronic infections (Costerton et al., 1999; James et al., 2008). A contributing factor to the persistence of these infections is the ability of cells within the biofilm to withstand antibiotic treatment. In *F. novicida* and LVS, biofilms have been shown to decrease the susceptibility of embedded bacteria to ciprofloxacin (Siebert et al., 2019, 2020), a first-line drug for post-exposure prophylaxis (Dennis et al., 2001). However, it was determined that ciprofloxacin-exposed biofilms readily entered a VBNC state (Siebert et al., 2020), providing yet an additional link between biofilm formation and a VBNC phenotype. Interestingly, Chung and colleagues demonstrated that biofilms formed by a *F. novicida chiA* mutant can be re-sensitized to an antibiotic it was previously resistant to if the ECM was enzymatically degraded (Chung et al., 2014). These studies highlight the potential health implications to consider if *F. tularensis* biofilm formation plays a role in human pathogenesis.

### Conclusion and future directions

Recent studies have demonstrated a link between phase variation and biofilm formation in *F. tularensis*. While the field appears to agree that both these phenotypes likely aid in environmental survival, many unanswered questions remain that would significantly advance our understanding of the mechanisms enabling survival and persistence, and even potentially help identify emerging threats in this genus of bacteria. Lastly, the focus of this review was on phase variation and biofilm formation; however, it is possible that multiple mechanisms and genetic pathways exist to control biofilm formation in *F. tularensis*. Understanding what role, if any, variation and biofilm plays in *F. tularensis* could allow for insights to develop better vaccines and therapeutics to prevent tularemia.

## Author contributions

KM and JB contributed to the conception and writing of this manuscript. All authors contributed to the article and approved the submitted version.

## Conflict of interest

The authors declare that the research was conducted in the absence of any commercial or financial relationships that could be construed as a potential conflict of interest.

## Publisher’s note

All claims expressed in this article are solely those of the authors and do not necessarily represent those of their affiliated organizations, or those of the publisher, the editors and the reviewers. Any product that may be evaluated in this article, or claim that may be made by its manufacturer, is not guaranteed or endorsed by the publisher.

## Author disclaimer

Opinions, interpretations, conclusions, and recommendations are those of the authors and are not necessarily endorsed by the US Army.

## References

[ref1] Abd H. Johansson T. Golovliov I. Sandstrom G. Forsman M. (2003). Survival and growth of Francisella tularensis in Acanthamoeba castellanii. Appl. Environ. Microbiol. 69, 600–606. doi: 10.1128/AEM.69.1.600-606.2003, PMID: 12514047 PMC152416

[ref2] Anthony L. D. Burke R. D. Nano F. E. (1991). Growth of Francisella spp. in rodent macrophages. Infect. Immun. 59, 3291–3296. doi: 10.1128/iai.59.9.3291-3296.1991, PMID: 1879943 PMC258167

[ref3] Apicella M. A. Post D. M. B. Fowler A. C. Jones B. D. Rasmussen J. A. Hunt J. R.. (2010). Identification, characterization and immunogenicity of an O-antigen capsular polysaccharide of Francisella tularensis. PLoS One 5:e11060. doi: 10.1371/journal.pone.0011060, PMID: 20625403 PMC2897883

[ref4] Backman S. Naslund J. Forsman M. Thelaus J. (2015). Transmission of tularemia from a water source by transstadial maintenance in a mosquito vector. Sci. Rep. 5:7793. doi: 10.1038/srep07793, PMID: 25609657 PMC4302321

[ref5] Bandara A. B. Champion A. E. Wang X. Berg G. Apicella M. A. McLendon M.. (2011). Isolation and mutagenesis of a capsule-like complex (CLC) from Francisella tularensis, and contribution of the CLC to F. tularensis virulence in mice. PLoS One 6:e19003. doi: 10.1371/journal.pone.0019003, PMID: 21544194 PMC3081320

[ref6] Ben Nasr A. Klimpel G. R. (2008). Subversion of complement activation at the bacterial surface promotes serum resistance and opsonophagocytosis of Francisella tularensis. J. Leukoc. Biol. 84, 77–85. doi: 10.1189/jlb.0807526, PMID: 18430786

[ref7] Burroughs A. L. H. R. Longanecker D. S. Meyer K. F. (1945). A field study of latent tularemia in rodents with a list of all known naturally infected vertebrates. J Infect Dis 76, 115–119. doi: 10.1093/infdis/76.2.115

[ref8] Champion A. E. Catanzaro K. C. F. Bandara A. B. Inzana T. J. (2019). Formation of the Francisella tularensis biofilm is affected by cell surface glycosylation, growth medium, and a glucan exopolysaccharide. Sci. Rep. 9:12252. doi: 10.1038/s41598-019-48697-x, PMID: 31439876 PMC6706388

[ref9] Chance T. Chua J. Toothman R. G. Ladner J. T. Nuss J. E. Raymond J. L.. (2017). A spontaneous mutation in kdsD, a biosynthesis gene for 3 deoxy-D-manno-Octulosonic acid, occurred in a ciprofloxacin resistant strain of Francisella tularensis and caused a high level of attenuation in murine models of tularemia. PLoS One 12:e0174106. doi: 10.1371/journal.pone.0174106, PMID: 28328947 PMC5362203

[ref10] Cherwonogrodzky J. W. Knodel M. H. Spence M. R. (1994). Increased encapsulation and virulence of Francisella tularensis live vaccine strain (LVS) by subculturing on synthetic medium. Vaccine 12, 773–775. doi: 10.1016/0264-410X(94)90284-4, PMID: 7975855

[ref11] Chung M. C. Dean S. Marakasova E. S. Nwabueze A. O. van Hoek M. L. (2014). Chitinases are negative regulators of Francisella novicida biofilms. PLoS One 9:e93119. doi: 10.1371/journal.pone.0093119, PMID: 24664176 PMC3963990

[ref12] Clay C. D. Soni S. Gunn J. S. Schlesinger L. S. (2008). Evasion of complement-mediated lysis and complement C3 deposition are regulated by Francisella tularensis lipopolysaccharide O antigen. J. Immunol. 181, 5568–5578. doi: 10.4049/jimmunol.181.8.5568, PMID: 18832715 PMC2782685

[ref13] Coburn J. Maier T. Casey M. Padmore L. Sato H. Frank D. W. (2015). Reproducible and quantitative model of infection of Dermacentor variabilis with the live vaccine strain of Francisella tularensis. Appl. Environ. Microbiol. 81, 386–395. doi: 10.1128/AEM.02917-14, PMID: 25362054 PMC4272728

[ref14] Costerton J. W. Stewart P. S. Greenberg E. P. (1999). Bacterial biofilms: a common cause of persistent infections. Science 284, 1318–1322. doi: 10.1126/science.284.5418.1318 10334980

[ref15] Cowley S. C. Myltseva S. V. Nano F. E. (1996). Phase variation in Francisella tularensis affecting intracellular growth, lipopolysaccharide antigenicity and nitric oxide production. Mol. Microbiol. 20, 867–874. doi: 10.1111/j.1365-2958.1996.tb02524.x, PMID: 8793882

[ref16] Cowley S. C. Myltseva S. V. Nano F. E. (1997). Suppression of Francisella tularensis growth in the rat by co-infection with F. novicida. FEMS Microbiol. Lett. 153, 71–74. doi: 10.1111/j.1574-6968.1997.tb10465.x, PMID: 9252574

[ref17] Dennis D. T. Inglesby T. V. Henderson D. A. Bartlett J. G. Ascher M. S. Eitzen E.. (2001). Tularemia as a biological weapon: medical and public health management. JAMA 285, 2763–2773. doi: 10.1001/jama.285.21.2763 11386933

[ref18] Durham-Colleran M. W. Verhoeven A. B. van Hoek M. L. (2010). Francisella novicida forms in vitro biofilms mediated by an orphan response regulator. Microb. Ecol. 59, 457–465. doi: 10.1007/s00248-009-9586-9, PMID: 19763680

[ref19] Eigelsbach H. T. Braun W. Herring R. D. (1951). Studies on the variation of bacterium tularense. J. Bacteriol. 61, 557–569. doi: 10.1128/jb.61.5.557-569.1951, PMID: 14832199 PMC386045

[ref20] Eigelsbach H. T. Downs C. M. (1961). Prophylactic effectiveness of live and killed tularemia vaccines. I. Production of vaccine and evaluation in the white mouse and Guinea pig. J. Immunol. 87, 415–425. 13889609

[ref21] El-Etr S. H. Margolis J. J. Monack D. Robison R. A. Cohen M. Moore E.. (2009). Francisella tularensis type a strains cause the rapid encystment of Acanthamoeba castellanii and survive in amoebal cysts for three weeks postinfection. Appl. Environ. Microbiol. 75, 7488–7500. doi: 10.1128/AEM.01829-09, PMID: 19820161 PMC2786426

[ref22] Eliasson H. Broman T. Forsman M. Back E. (2006). Tularemia: current epidemiology and disease management. Infect. Dis. Clin. North Am. 20, 289–311. doi: 10.1016/j.idc.2006.03.002, PMID: 16762740

[ref23] Ellis J. Oyston P. C. Green M. Titball R. W. (2002). Tularemia. Clin. Microbiol. Rev. 15, 631–646. doi: 10.1128/CMR.15.4.631-646.2002, PMID: 12364373 PMC126859

[ref24] Forestal C. A. Malik M. Catlett S. V. Savitt A. G. Benach J. L. Sellati T. J.. (2007). Francisella tularensis has a significant extracellular phase in infected mice. J Infect Dis 196, 134–137. doi: 10.1086/518611, PMID: 17538893

[ref25] Forsman M. Henningson E. W. Larsson E. Johansson T. Sandstrom G. (2000). Francisella tularensis does not manifest virulence in viable but non-culturable state. FEMS Microbiol. Ecol. 31, 217–224. doi: 10.1111/j.1574-6941.2000.tb00686.x 10719202

[ref26] Foshay L. (1932). Induction of Avirulence in Pasteurella Tularensis. J Infect Dis 51, 280–285. doi: 10.1093/infdis/51.2.280

[ref27] Francis E. (1919). Deer-Fly fever, or Pahvant Valley plague: a disease of man of hitherto unknown etiology. Public Health Rep. 34, 2061–2062. doi: 10.2307/457530619314686

[ref28] Freudenberger Catanzaro K. C. Inzana T. J. (2020). The Francisella tularensis polysaccharides: what is the real capsule? Microbiol. Mol. Biol. Rev. 84:e00065-19. doi: 10.1128/MMBR.00065-19, PMID: 32051235 PMC7018499

[ref29] Galperin M. Y. Nikolskaya A. N. Koonin E. V. (2001). Novel domains of the prokaryotic two-component signal transduction systems. FEMS Microbiol. Lett. 203, 11–21. doi: 10.1111/j.1574-6968.2001.tb10814.x, PMID: 11557134

[ref30] Goethert H. K. Shani I. Telford S. R. (2004). Genotypic diversity of Francisella tularensis infecting Dermacentor variabilis ticks on Martha's Vineyard, Massachusetts. J. Clin. Microbiol. 42, 4968–4973. doi: 10.1128/JCM.42.11.4968-4973.2004, PMID: 15528681 PMC525218

[ref31] Goethert H. K. Telford S. R. (2009). Nonrandom distribution of vector ticks (Dermacentor variabilis) infected by Francisella tularensis. PLoS Pathog. 5:e1000319. doi: 10.1371/journal.ppat.1000319, PMID: 19247435 PMC2642597

[ref32] Golovliov I. Bäckman S. Granberg M. Salomonsson E. Lundmark E. Näslund J.. (2021). Long-term survival of virulent tularemia pathogens outside a host in conditions that mimic natural aquatic environments. Appl. Environ. Microbiol. 87:e02713-20. doi: 10.1128/AEM.02713-20, PMID: 33397692 PMC8104992

[ref33] Gunn J. S. Ernst R. K. (2007). The structure and function of Francisella lipopolysaccharide. Ann. N. Y. Acad. Sci. 1105, 202–218. doi: 10.1196/annals.1409.006, PMID: 17395723 PMC2742961

[ref34] Hall-Stoodley L. Costerton J. W. Stoodley P. (2004). Bacterial biofilms: from the natural environment to infectious diseases. Nat. Rev. Microbiol. 2, 95–108. doi: 10.1038/nrmicro821 15040259

[ref35] Hall-Stoodley L. Stoodley P. (2009). Evolving concepts in biofilm infections. Cell. Microbiol. 11, 1034–1043. doi: 10.1111/j.1462-5822.2009.01323.x, PMID: 19374653

[ref36] Hartley G. Taylor R. Prior J. Newstead S. Hitchen P. G. Morris H. R.. (2006). Grey variants of the live vaccine strain of Francisella tularensis lack lipopolysaccharide O-antigen, show reduced ability to survive in macrophages and do not induce protective immunity in mice. Vaccine 24, 989–996. doi: 10.1016/j.vaccine.2005.08.075, PMID: 16257097

[ref37] Hathroubi S. Hancock M. A. Bossé J. T. Langford P. R. Tremblay Y. D. N. Labrie J.. (2016). Surface polysaccharide mutants reveal that absence of O antigen reduces biofilm formation of Actinobacillus pleuropneumoniae. Infect. Immun. 84, 127–137. doi: 10.1128/IAI.00912-15, PMID: 26483403 PMC4694004

[ref38] Hickman J. W. Tifrea D. F. Harwood C. S. (2005). A chemosensory system that regulates biofilm formation through modulation of cyclic diguanylate levels. Proc. Natl. Acad. Sci. U. S. A. 102, 14422–14427. doi: 10.1073/pnas.0507170102, PMID: 16186483 PMC1234902

[ref39] Hinnebusch B. J. Erickson D. L. (2008). Yersinia pestis biofilm in the flea vector and its role in the transmission of plague. Curr. Top. Microbiol. Immunol. 322, 229–248. doi: 10.1007/978-3-540-75418-3_11 18453279 PMC3727414

[ref40] Holland K. M. Rosa S. J. Kristjansdottir K. Wolfgeher D. Franz B. J. Zarrella T. M.. (2017). Differential growth of Francisella tularensis, which alters expression of virulence factors, dominant antigens, and surface-carbohydrate synthases, governs the apparent virulence of Ft SchuS4 to immunized animals. Front. Microbiol. 8:1158. doi: 10.3389/fmicb.2017.01158, PMID: 28690600 PMC5479911

[ref41] Hood A. M. (1977). Virulence factors of Francisella tularensis. J. Hyg. 79, 47–60. doi: 10.1017/S0022172400052840, PMID: 267668 PMC2129918

[ref42] Hopla C. E. (1974). The ecology of tularemia. Adv. Vet. Sci. Comp. Med. 18, 25–53. PMID: 4419176

[ref43] Hornick R. B. Eigelsbach H. T. (1966). Aerogenic immunization of man with live tularemia vaccine. Bacteriol. Rev. 30, 532–538. doi: 10.1128/br.30.3.532-538.1966, PMID: 5917334 PMC378235

[ref44] James G. A. Swogger E. Wolcott R. Pulcini E. L. Secor P. Sestrich J.. (2008). Biofilms in chronic wounds. Wound Repair Regen. 16, 37–44. doi: 10.1111/j.1524-475X.2007.00321.x 18086294

[ref45] Jarrett C. O. Deak E. Isherwood K. E. Oyston P. C. Fischer E. R. Whitney A. R.. (2004). Transmission of Yersinia pestis from an infectious biofilm in the flea vector. J Infect Dis 190, 783–792. doi: 10.1086/422695, PMID: 15272407

[ref46] Jellison W. Tularemia in North America. (University of Montana Foundation, University of Montana; (1974).

[ref47] Jenal U. Reinders A. Lori C. (2017). Cyclic di-GMP: second messenger extraordinaire. Nat. Rev. Microbiol. 15, 271–284. doi: 10.1038/nrmicro.2016.190, PMID: 28163311

[ref48] Jones B. D. Faron M. Rasmussen J. A. Fletcher J. R. (2014). Uncovering the components of the Francisella tularensis virulence stealth strategy. Front. Cell. Infect. Microbiol. 4:32. doi: 10.3389/fcimb.2014.00032 24639953 PMC3945745

[ref49] Kim T. H. Pinkham J. T. Heninger S. J. Chalabaev S. Kasper D. L. (2012). Genetic modification of the O-polysaccharide of Francisella tularensis results in an avirulent live attenuated vaccine. J Infect Dis 205, 1056–1065. doi: 10.1093/infdis/jir620, PMID: 21969334 PMC3295600

[ref50] Kim Y. S. Ryu J. H. Han S. J. Choi K. H. Nam K. B. Jang I. H.. (2000). Gram-negative bacteria-binding protein, a pattern recognition receptor for lipopolysaccharide and beta-1,3-glucan that mediates the signaling for the induction of innate immune genes in Drosophila melanogaster cells. J. Biol. Chem. 275, 32721–32727. doi: 10.1074/jbc.M003934200, PMID: 10827089

[ref51] Kingry L. C. Petersen J. M. (2014). Comparative review of Francisella tularensis and Francisella novicida. Front. Cell. Infect. Microbiol. 4:35. doi: 10.3389/fcimb.2014.00035 24660164 PMC3952080

[ref52] Li J. Ryder C. Mandal M. Ahmed F. Azadi P. Snyder D. S.. (2007). Attenuation and protective efficacy of an O-antigen-deficient mutant of Francisella tularensis LVS. Microbiology 153, 3141–3153. doi: 10.1099/mic.0.2007/006460-0, PMID: 17768257

[ref53] Lindemann S. R. Peng K. Long M. E. Hunt J. R. Apicella M. A. Monack D. M.. (2011). Francisella tularensis Schu S4 O-antigen and capsule biosynthesis gene mutants induce early cell death in human macrophages. Infect. Immun. 79, 581–594. doi: 10.1128/IAI.00863-10, PMID: 21078861 PMC3028865

[ref54] Lundstrom J. O. Andersson A. C. Backman S. Schafer M. L. Forsman M. Thelaus J.. (2011). Transstadial transmission of Francisella tularensis holarctica in mosquitoes, Sweden. Emerg. Infect. Dis. 17, 794–799. doi: 10.3201/eid1705.100426, PMID: 21529386 PMC3321753

[ref55] Mahajan U. V. Gravgaard J. Turnbull M. Jacobs D. B. McNealy T. L. (2011). Larval exposure to Francisella tularensis LVS affects fitness of the mosquito Culex quinquefasciatus. FEMS Microbiol. Ecol. 78, 520–530. doi: 10.1111/j.1574-6941.2011.01182.x, PMID: 22066999

[ref56] Mani R. J. Metcalf J. A. Clinkenbeard K. D. (2015). Amblyomma americanum as a bridging vector for human infection with Francisella tularensis. PLoS One 10:e0130513. doi: 10.1371/journal.pone.0130513, PMID: 26121137 PMC4486451

[ref57] Margolis J. J. el-Etr S. Joubert L. M. Moore E. Robison R. Rasley A.. (2010). Contributions of Francisella tularensis subsp. novicida chitinases and sec secretion system to biofilm formation on chitin. Appl. Environ. Microbiol. 76, 596–608. doi: 10.1128/AEM.02037-09, PMID: 19948864 PMC2805214

[ref58] McCaig W. D. Koller A. Thanassi D. G. (2013). Production of outer membrane vesicles and outer membrane tubes by Francisella novicida. J. Bacteriol. 195, 1120–1132. doi: 10.1128/JB.02007-12, PMID: 23264574 PMC3592013

[ref59] McLendon M. K. Apicella M. A. Allen L. A. (2006). Francisella tularensis: taxonomy, genetics, and Immunopathogenesis of a potential agent of biowarfare. Annu. Rev. Microbiol. 60, 167–185. doi: 10.1146/annurev.micro.60.080805.142126, PMID: 16704343 PMC1945232

[ref60] Mirzaei R. Mohammadzadeh R. Sholeh M. Karampoor S. Abdi M. Dogan E.. (2020). The importance of intracellular bacterial biofilm in infectious diseases. Microb. Pathog. 147:104393. doi: 10.1016/j.micpath.2020.104393, PMID: 32711113

[ref61] Mlynek K. D. Lopez C. T. Fetterer D. P. Williams J. A. Bozue J. A. (2021). Phase variation of LPS and capsule is responsible for stochastic biofilm formation in Francisella tularensis. Front. Cell. Infect. Microbiol. 11:808550. doi: 10.3389/fcimb.2021.808550 35096655 PMC8795689

[ref62] Murphy K. Park A. J. Hao Y. Brewer D. Lam J. S. Khursigara C. M. (2014). Influence of O polysaccharides on biofilm development and outer membrane vesicle biogenesis in Pseudomonas aeruginosa PAO1. J. Bacteriol. 196, 1306–1317. doi: 10.1128/JB.01463-13, PMID: 24464462 PMC3993350

[ref63] Nakao R. Ramstedt M. Wai S. N. Uhlin B. E. (2012). Enhanced biofilm formation by Escherichia coli LPS mutants defective in Hep biosynthesis. PLoS One 7:e51241. doi: 10.1371/journal.pone.0051241, PMID: 23284671 PMC3532297

[ref64] Nakao R. Senpuku H. Watanabe H. (2006). Porphyromonas gingivalis galE is involved in lipopolysaccharide O-antigen synthesis and biofilm formation. Infect. Immun. 74, 6145–6153. doi: 10.1128/IAI.00261-06, PMID: 16954395 PMC1695533

[ref65] Parker R. R. Spencer R. R. Francis E. (1924). Tularaemia in ticks of the species Dermacentor Andersoni stiles in the Bitterroot Valley. Mont. Public Health Rep. 39, 1057–1073. doi: 10.2307/4577151

[ref66] Parker R. R. Steinhaus E. A. Kohls G. M. Jellison W. L. (1951). Contamination of natural waters and mud with Pasteurella tularensis and tularemia in beavers and muskrats in the northwestern United States. Bull Natl. Inst. Health 193, 1–161. PMID: 14869929

[ref67] Pavkova I. Klimentova J. Bavlovic J. Horcickova L. Kubelkova K. Vlcak E.. (2021). Francisella tularensis outer membrane vesicles participate in the early phase of interaction with macrophages. Front. Microbiol. 12:748706. doi: 10.3389/fmicb.2021.748706, PMID: 34721352 PMC8554293

[ref68] Prior J. L. Prior R. G. Hitchen P. G. Diaper H. Griffin K. F. Morris H. R.. (2003). Characterization of the O antigen gene cluster and structural analysis of the O antigen of Francisella tularensis subsp. tularensis. J. Med. Microbiol. 52, 845–851. doi: 10.1099/jmm.0.05184-0, PMID: 12972577

[ref69] Ransmeier J. C. (1943). The reaction of the Chick embryo to virulent and nonvirulent strains of Bact. Tularense. J Infect Dis 72, 86–90. doi: 10.1093/infdis/72.1.86

[ref70] Rasmussen J. A. Fletcher J. R. Long M. E. Allen L. A. Jones B. D. (2015). Characterization of Francisella tularensis Schu S4 mutants identified from a transposon library screened for O-antigen and capsule deficiencies. Front. Microbiol. 6:338. doi: 10.3389/fmicb.2015.00338 25999917 PMC4419852

[ref71] Rasmussen J. A. Post D. M. B. Gibson B. W. Lindemann S. R. Apicella M. A. Meyerholz D. K.. (2014). Francisella tularensis Schu S4 lipopolysaccharide core sugar and O-antigen mutants are attenuated in a mouse model of tularemia. Infect. Immun. 82, 1523–1539. doi: 10.1128/IAI.01640-13, PMID: 24452684 PMC3993386

[ref72] Raynaud C. Meibom K. L. Lety M. A. Dubail I. Candela T. Frapy E.. (2007). Role of the wbt locus of Francisella tularensis in lipopolysaccharide O-antigen biogenesis and pathogenicity. Infect. Immun. 75, 536–541. doi: 10.1128/IAI.01429-06, PMID: 17030571 PMC1828372

[ref73] Rohmer L. Fong C. Abmayr S. Wasnick M. Larson Freeman T. Radey M.. (2007). Comparison of Francisella tularensis genomes reveals evolutionary events associated with the emergence of human pathogenic strains. Genome Biol. 8:R102. doi: 10.1186/gb-2007-8-6-r102, PMID: 17550600 PMC2394750

[ref74] Rosenberg R. Lindsey N. P. Fischer M. Gregory C. J. Hinckley A. F. Mead P. S.. (2018). Vital signs: trends in reported Vectorborne disease cases - United States and territories, 2004-2016. MMWR Morb. Mortal. Wkly Rep. 67, 496–501. doi: 10.15585/mmwr.mm6717e1, PMID: 29723166 PMC5933869

[ref75] Sandstrom G. Lofgren S. Tarnvik A. (1988). A capsule-deficient mutant of Francisella tularensis LVS exhibits enhanced sensitivity to killing by serum but diminished sensitivity to killing by polymorphonuclear leukocytes. Infect. Immun. 56, 1194–1202. doi: 10.1128/iai.56.5.1194-1202.1988, PMID: 3356465 PMC259783

[ref76] Sandstrom G. Sjostedt A. Johansson T. Kuoppa K. Williams J. C. (1992). Immunogenicity and toxicity of lipopolysaccharide from Francisella tularensis LVS. FEMS Microbiol. Immunol. 5, 201–210. doi: 10.1111/j.1574-6968.1992.tb05902.x, PMID: 1419118

[ref77] Saslaw S. Eigelsbach H. T. Prior J. A. Wilson H. E. Carhart S. (1961). Tularemia vaccine study. II. Respiratory challenge. Arch. Intern. Med. 107, 702–714. doi: 10.1001/archinte.1961.03620050068007, PMID: 13746667

[ref79] Siebert C. Lindgren H. Ferré S. Villers C. Boisset S. Perard J.. (2019). Francisella tularensis: FupA mutation contributes to fluoroquinolone resistance by increasing vesicle secretion and biofilm formation. Emerg. Microbes Infect. 8, 808–822. doi: 10.1080/22221751.2019.1615848, PMID: 31164053 PMC6566608

[ref80] Siebert C. Villers C. Pavlou G. Touquet B. Yakandawala N. Tardieux I.. (2020). Francisella novicida and F. philomiragia biofilm features conditioning fitness in spring water and in presence of antibiotics. PLoS One 15:e0228591. doi: 10.1371/journal.pone.0228591, PMID: 32023304 PMC7001994

[ref81] Simm R. Morr M. Kader A. Nimtz M. Romling U. (2004). GGDEF and EAL domains inversely regulate cyclic di-GMP levels and transition from sessility to motility. Mol. Microbiol. 53, 1123–1134. doi: 10.1111/j.1365-2958.2004.04206.x, PMID: 15306016

[ref82] Sjodin A. Svensson K. Ohrman C. Ahlinder J. Lindgren P. Duodu S.. (2012). Genome characterisation of the genus Francisella reveals insight into similar evolutionary paths in pathogens of mammals and fish. BMC Genomics 13:268. doi: 10.1186/1471-2164-13-268, PMID: 22727144 PMC3485624

[ref83] Soni S. Ernst R. K. Muszynski A. Mohapatra N. P. Perry M. B. Vinogradov E.. (2010). Francisella tularensis blue-gray phase variation involves structural modifications of lipopolysaccharide o-antigen, core and lipid a and affects intramacrophage survival and vaccine efficacy. Front. Microbiol. 1:129. doi: 10.3389/fmicb.2010.00129 21687776 PMC3109528

[ref84] Sorokin V. M. Pavlovich N. V. Prozorova L. A. (1996). Francisella tularensis resistance to bactericidal action of normal human serum. FEMS Immunol. Med. Microbiol. 13, 249–252. doi: 10.1111/j.1574-695X.1996.tb00246.x, PMID: 8861038

[ref85] Tamayo R. Pratt J. T. Camilli A. (2007). Roles of cyclic diguanylate in the regulation of bacterial pathogenesis. Annu. Rev. Microbiol. 61, 131–148. doi: 10.1146/annurev.micro.61.080706.093426, PMID: 17480182 PMC2776827

[ref86] Telford S. R. Goethert H. K. (2020). Ecology of Francisella tularensis. Annu. Rev. Entomol. 65, 351–372. doi: 10.1146/annurev-ento-011019-025134, PMID: 31600457 PMC8300880

[ref87] Thelaus J. Andersson A. Mathisen P. Forslund A. L. Noppa L. Forsman M. (2009). Influence of nutrient status and grazing pressure on the fate of Francisella tularensis in lake water. FEMS Microbiol. Ecol. 67, 69–80. doi: 10.1111/j.1574-6941.2008.00612.x, PMID: 19120459

[ref88] Thomas R. M. Titball R. W. Oyston P. C. F. Griffin K. Waters E. Hitchen P. G.. (2007). The immunologically distinct O antigens from Francisella tularensis subspecies tularensis and Francisella novicida are both virulence determinants and protective antigens. Infect. Immun. 75, 371–378. doi: 10.1128/IAI.01241-06, PMID: 17074846 PMC1828428

[ref89] Tully B. G. Huntley J. F. (2020). Mechanisms affecting the acquisition, persistence and transmission of Francisella tularensis in ticks. Microorganisms 8:1639. doi: 10.3390/microorganisms8111639, PMID: 33114018 PMC7690693

[ref90] Twine S. M. Vinogradov E. Lindgren H. Sjostedt A. Conlan J. W. (2012). Roles for wbtC, wbtI, and kdtA genes in lipopolysaccharide biosynthesis, protein glycosylation, virulence, and immunogenicity in Francisella tularensis2 strain SCHU S4. Pathogens 1, 12–29. doi: 10.3390/pathogens1010012, PMID: 25152813 PMC4141488

[ref91] van Hoek M. L. (2013). Biofilms: an advancement in our understanding of Francisella species. Virulence 4, 833–846. doi: 10.4161/viru.27023, PMID: 24225421 PMC3925715

[ref92] Vinogradov E. Conlan W. J. Gunn J. S. Perry M. B. (2004). Characterization of the lipopolysaccharide O-antigen of Francisella novicida (U112). Carbohydr. Res. 339, 649–654. doi: 10.1016/j.carres.2003.12.013, PMID: 15013402

[ref93] Vinogradov E. Perry M. B. Conlan J. W. (2002). Structural analysis of Francisella tularensis lipopolysaccharide. Eur. J. Biochem. 269, 6112–6118. doi: 10.1046/j.1432-1033.2002.03321.x 12473106

[ref94] Vinogradov E. V. Shashkov A. S. Knirel Y. A. Kochetkov N. K. Tochtamysheva N. V. Averin S. F.. (1991). Structure of the O-antigen of Francisella tularensis strain 15. Carbohydr. Res. 214, 289–297. PMID: 1769021 10.1016/0008-6215(91)80036-m

[ref95] Wang X. Ribeiro A. A. Guan Z. Abraham S. N. Raetz C. R. (2007). Attenuated virulence of a Francisella mutant lacking the lipid a 4′-phosphatase. Proc. Natl. Acad. Sci. U. S. A. 104, 4136–4141. doi: 10.1073/pnas.0611606104, PMID: 17360489 PMC1820721

[ref96] Weiss D. S. Brotcke A. Henry T. Margolis J. J. Chan K. Monack D. M. (2007). In vivo negative selection screen identifies genes required for Francisella virulence. Proc. Natl. Acad. Sci. U. S. A. 104, 6037–6042. doi: 10.1073/pnas.0609675104, PMID: 17389372 PMC1832217

[ref97] Wildschutte H. Wolfe D. M. Tamewitz A. Lawrence J. G. (2004). Protozoan predation, diversifying selection, and the evolution of antigenic diversity in salmonella. Proc. Natl. Acad. Sci. U. S. A. 101, 10644–10649. doi: 10.1073/pnas.0404028101, PMID: 15247413 PMC489988

[ref98] Xia X. You M. Rao X. J. Yu X. Q. (2018). Insect C-type lectins in innate immunity. Dev. Comp. Immunol. 83, 70–79. doi: 10.1016/j.dci.2017.11.020 29198776

[ref99] Yu J. J. Raulie E. K. Murthy A. K. Guentzel M. N. Klose K. E. Arulanandam B. P. (2008). The presence of infectious extracellular Francisella tularensis subsp. novicida in murine plasma after pulmonary challenge. Eur. J. Clin. Microbiol. Infect. Dis. 27, 323–325. doi: 10.1007/s10096-007-0434-x, PMID: 18087734

[ref100] Zarrella T. M. Singh A. Bitsaktsis C. Rahman T. Sahay B. Feustel P. J.. (2011). Host-adaptation of Francisella tularensis alters the bacterium's surface-carbohydrates to hinder effectors of innate and adaptive immunity. PLoS One 6:e22335. doi: 10.1371/journal.pone.0022335, PMID: 21799828 PMC3142145

[ref101] Zellner B. Huntley J. F. (2019). Ticks and tularemia: do we know what we Don't know? Front. Cell. Infect. Microbiol. 9:146. doi: 10.3389/fcimb.2019.00146, PMID: 31139576 PMC6517804

[ref102] Zimmer A. L. Thoden J. B. Holden H. M. (2014). Three-dimensional structure of a sugar N-formyltransferase from Francisella tularensis. Protein Sci. 23, 273–283. doi: 10.1002/pro.2409, PMID: 24347283 PMC3945835

[ref103] Zogaj X. Wyatt G. C. Klose K. E. (2012). Cyclic di-GMP stimulates biofilm formation and inhibits virulence of Francisella novicida. Infect. Immun. 80, 4239–4247. doi: 10.1128/IAI.00702-12, PMID: 22988021 PMC3497427

